# Genome-Wide Disruption of Gene Expression in Allopolyploids but Not Hybrids of Rice Subspecies

**DOI:** 10.1093/molbev/msu085

**Published:** 2014-02-27

**Authors:** Chunming Xu, Yan Bai, Xiuyun Lin, Na Zhao, Lanjuan Hu, Zhiyun Gong, Jonathan F. Wendel, Bao Liu

**Affiliations:** ^1^Key Laboratory of Molecular Epigenetics of the Ministry of Education (MOE), Northeast Normal University, Changchun, China; ^2^Department of Ecology, Evolution and Organismal Biology, Iowa State University; ^3^Jilin Academy of Agricultural Sciences, Changchun, China; ^4^Faculty of Agronomy, Jilin Agricultural University, Changchun, China; ^5^Agricultural College, Yangzhou University, Yangzhou, China

**Keywords:** hybridization, polyploidization, homoeologous expression, evolution of gene regulation, *Oryza sativa* L

## Abstract

Hybridization and polyploidization are prominent processes in plant evolution. Hybrids and allopolyploids typically exhibit radically altered gene expression patterns relative to their parents, a phenomenon termed “transcriptomic shock.” To distinguish the effects of hybridization from polyploidization on coregulation of divergent alleles, we analyzed expression of parental copies (homoeologs) of 11,608 genes using RNA-seq-based transcriptome profiling in reciprocal hybrids and tetraploids constructed from subspecies *japonica* and *indica* of Asian rice (*Oryza sativa* L.). The diploid hybrids and their derived allopolyploids differ dramatically in morphology, despite having the same suite of genes and genic proportions. Allelic and homoeolog-specific transcripts were unequivocally diagnosed in the hybrids and tetraploids based on parent-specific SNPs. Compared with the in silico hybrid (parental mix), the range of progenitor expression divergence was significantly reduced in both reciprocally generated F1 hybrids, presumably due to the ameliorating effects of a common *trans* environment on divergent *cis*-factors. In contrast, parental expression differences were greatly elaborated at the polyploid level, which we propose is a consequence of stoichiometric disruptions associated with the numerous chromosomal packaging and volumetric changes accompanying nascent polyploidy. We speculate that the emergent property of “whole genome doubling” has repercussions that reverberate throughout the transcriptome and downstream, ultimately generating altered phenotypes. This perspective may yield insight into the nature of adaptation and the origin of evolutionary novelty accompanying polyploidy.

## Introduction

Polyploidy, or whole genome duplication (WGD), has played a pervasive role in plant evolution (Wendel 2000; Adams 2007; Chen 2007; Doyle et al. 2008), with all angiosperms having experienced several WGD episodes in their evolutionary histories (Soltis et al. 2009; Jiao et al. 2011). Allopolyploidy in particular is thought to have played a significant role in plant diversification and remains an important speciation process today (Soltis et al. 2009; Chelaifa et al. 2010; Ainouche et al. 2012; Feldman and Levy 2012; Soltis et al. 2012; Abbott et al. 2013). It has been proposed that the ability to rapidly generate genetic and/or expression diversity is essential for newly formed polyploids to survive and establish (Otto 2007). This should be especially relevant under ecological conditions involving a changing environment or novel niche for colonization by nascent polyploid (Fawcett et al. 2009; Abbott et al. 2013). An array of studies in diverse plant taxa have documented that polyploidy and especially allopolyploidy may induce a cascade of rapid genomic and gene expression changes (Wendel 2000; Levy and Feldman 2004; Adams and Wendel 2005; Adams 2007; Chen 2007; Otto 2007; Doyle et al. 2008; Soltis et al. 2009; Feldman and Levy 2012; Abbott et al. 2013; Buggs 2013). In particular, rapid changes in gene expression, as reflected by expression nonadditivity from that predicted by the null hypothesis of parental average (Gianinetti 2013), appear to be widespread in newly formed allopolyploids, a phenomenon that has been termed “transcriptomic shock” (Hegarty et al. 2006; Buggs et al. 2011).

In cases of allopolyploidy, both hybridization and WGD may initiate transcriptomic shock. However, hybridization and WGD are often entangled in natural settings, and therefore, synthetic F1 hybrids and polyploids have been used to deconvolute the effects of the two events on genome expression (Flagel et al. 2008; Hegarty 2011). In this respect, the only case where both naturally occurring, extant F1 hybrids and their derived allopolyploids have been studied is in *Spartina* (Poaceae), including F1 hybrids (*S.* × *townsendii*) and allopolyploids (*Spartina anglica*) (Ainouche et al. 2004). With regard to the studied synthetic F1 hybrids and allopolyploids conducted in various plant systems, highly variable results have been obtained with respect to the impacts of the two events on locus-specific or genome-wide gene expression patterns in different plant taxa, including large effects of hybridization but negligible effects of WGD in *Arabidopsis* (Wang et al. 2006; Chen 2007), similar effects of both hybridization and WGD in cotton (Flagel et al. 2008), amelioration of transcriptomic shock by WGD in *Senecio* (Hegarty et al. 2006), to antagonistic effects of hybridization and WGD on tissue-specific homoeologous expression in *Tragopogon* (Buggs et al. 2011). In the case of *Spartina*, different effects of hybridization and WGD on global gene expression patterns were observed based on microarray analysis: while both transgressive gene expression and maternal expression dominance were induced by hybridization, WGD caused additional genes showing transgressive expression but attenuated the extent of maternal expression dominance (Chelaifa et al. 2010). Several studies have addressed the question of homoeolog expression levels in hybrids and allopolyploids for a limited number of genes (Koh et al. 2010; Buggs et al. 2011; Dong and Adams 2011; Combes et al. 2012), and also more globally using genome-wide screens (Bancroft et al. 2011; Higgins et al. 2012; Combes et al. 2013). In cotton, Yoo et al. (2013), for example, detailed the separate as well as combined effects of hybridization and WGD on biased expression of parental homoeologs and their correlation with expression level dominance, another prominent phenomenon commonly associated with allopolyploidy (Yoo et al. 2013). It was found that both hybridization and WGD contribute to homoeologous expression divergence, and that the effects become more prominent in natural polyploid cotton species than in synthetic polyploids with the same genome compositions (Yoo et al. 2013). It is clear that further investigations in additional plant systems are needed to enhance our understanding of the scope and scale of the transcriptomic alterations accompanying hybridization and polyploidy, as well as its implications with respect to phenotype and function.

Here, we take a step in this direction using a model system involving the two subspecies of Asian rice (*Oryza sativa* L.), subsp. *japonica* and subsp. *indica*, each having been domesticated from a common wild progenitor, *O. rufipogon*, approximately 9,000 years ago (Kovach et al. 2007; Huang et al. 2012). Following initial domestication, each of these taxa was selected for adaptation to distinct ecological conditions. Although multiple rounds of reciprocal introgression between *japonica* and *indica* likely have occurred at various times (Yang et al. 2012), extensive genetic and genomic divergence still are evident in the two subspecies. For example, genome-wide full-length cDNA analysis revealed that 45.8% of the *indica-japonica* cDNA pairs harbor differences at the protein level due to nonsynonymous single-nucleotide polymorphisms (SNPs), insertions or deletions, and segment variations between the two subspecies (Liu et al. 2007). For purposes of the present analysis, we took advantage of the availability of high-quality genome sequences for each subspecies (Goff et al. 2002; Yu et al. 2002; International Rice Genome Sequencing Project 2005). Here, we conducted global transcriptomic profiling of pure-lines of each Asian rice subspecies, their reciprocal F1 hybrids, and synthetic tetraploids, to gain insight into the impacts of hybridization and WGD on genome-wide expression patterns. We report on the construction of this useful experimental resource using homozygous individual plants of cultivars Nipponbare (*japonica*) and 93-11 (*indica*) as crossing parents and the results of deep RNA-seq-based transcriptome analysis of parental homoeologous gene expression. Our results reveal the distinct and in some respects opposite effects of genome merger and genome doubling on expression profiles in the hybrids and allopolyploids.

## Results

### Rice Inter-Subspecies Tetraploids Have a Stable Euploid Karyotype but Distinct Phenotypes

**Fig. 1.**
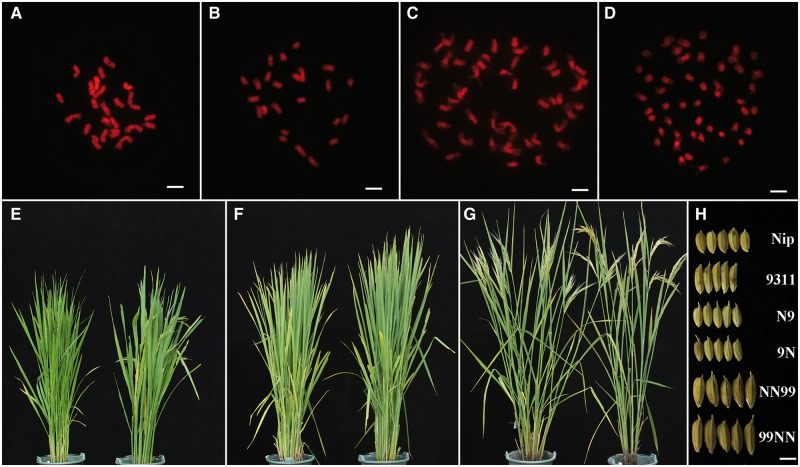
Cytological and phenotypic characteristics of the two diploid parental genotypes, Nipponbare and 93-11, representing the two subspecies, *japonica* and *indica*, respectively, of Asian rice (*Oryza sativa* L.), their reciprocal F1 hybrids and S1 tetraploids. Somatic chromosome numbers of the two parental genotypes, Nipponbare (*A*) and 93-11 (*B*), and the reciprocal tetraploids (*C* and *D*) are shown, which are 2*n* = 24 and 48, respectively. (*E–G*) Overall morphology of mature plants of the parental genotypes, Nipponbare (*E*, left) and 93-11 (*E*, right), their reciprocal F1 hybrids, N9 (*F*, left) and 9N (*F*, right), and the reciprocal tetraploids, NN99 (*G*, left) and 99NN (*G*, right). (*H*) Typical grain morphology of parents, hybrids, and tetraploids. Scale bars in (*A–D*) are 5 μm and in *H* is 0.5 cm.

Previous studies have documented significant vegetative growth vigor (biomass heterosis) but a high degree of sterility in the reciprocal F1 hybrids of the Asian rice (*Oryza sativa* L.) subspecies *japonica* (cv. Nipponbare) and *indica* (cv. 93-11), as well as globally altered gene expression relative to that expected from parental additivity (He et al. 2010; Ouyang et al. 2010; Chodavarapu et al. 2012). The genomic, transcriptomic, and phenotypic consequences of WGD resulting from these reciprocal hybrids, that is, tetraploidy, has remained unexplored. Here, we found that tetraploids derived from the reciprocal F1 hybrids of Nipponbare and 93-11 via colchicine-induced WGD are predominantly stable euploids (2*n* = 48) at the whole chromosomal level (fig. 1, upper panel), based on chromosome counting of >100 random individual plants at the S1 generation and its selfed progenies, in which >90 plants of both reciprocal tetraploids contained a chromosome number of 48 (supplementary fig. S1, Supplementary Material online). The euploid tetraploids largely retained the biomass heterosis of the F1 hybrids but showed differences in several morphological traits, including increased height, earlier flowering, improved fertility, and enlarged panicle and seed sizes (fig.1, lower panel). No conspicuous phenotypic difference was noted between the reciprocal F1 hybrids, consistent with previous observations (He et al. 2010), or between the reciprocal tetraploids (fig.1, lower panel). The modified phenotypes in the immediate tetraploids, versus hybrids, implies altered gene expression between these two ploidy levels, given that they share the same merged parental genomes.

### Hybrids and Tetraploids Show Distinct Spectra of Genome-Wide Parental Homoeologous Expression Divergence

To understand expression changes of parental homoeologs in the reciprocal F1 hybrids and tetraploids, we performed deep-coverage RNA-seq on immature panicle tissue of the reciprocal F1 hybrids (N9 and 9N) and their reciprocal tetraploids (NN99 and 99NN), along with their diploid parents, Nipponbare and 93-11. An in silico hybrid was constructed by mixing the diploid parental RNA-seq data at a ratio of 1:1, to reflect accumulated gene expression divergence of the two parents (*japonica* and *indica*) since their domestication from a common accession of their wild progenitor, *O. rufipogon*, and/or inherited standing polymorphisms of different accessions of the wild progenitor involved (Huang et al. 2012).

**Fig. 2.**
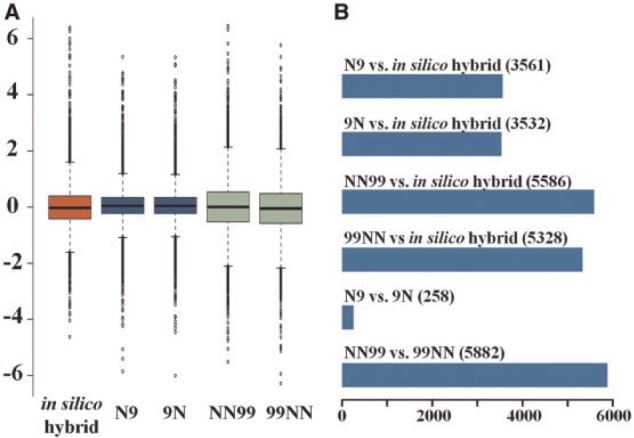
RNA-seq-based whole-genome expression spectra of parental (allelic, at the diploid level) and homoeologous (at the polyploid level) expression divergence for a total of 11,608 genes in the in silico hybrid, reciprocal F1 hybrids, and reciprocal tetraploids, revealed by a log2-transformed boxplot (*A*), and the numbers of genes showing differential expression of parental homoeologs in each pairwise comparison (*B*).

In total, 11,608 homoeologous genes harboring unequivocal homolog-diagnostic SNPs were selected for analysis (see Materials and Methods, supplementary table S1, Supplementary Material online). We found that 5,823 out of the 11,608 genes are differentially expressed between the two parental cultivars, Nipponbare and 93-11, representing the Asian rice subspecies, *japonica* and *indica*, respectively (ratio of parental homoeolog expression ≠ 1:1, based on binominal test, followed by false discovery rate (FDR) adjustment). Log-transformed relative expression ratios of the 11,608 homoeologous genes in each of the plant samples, including the in silico hybrid, N9, 9N, NN99, and 99NN, are illustrated with a boxplot (fig. 2*A*). Compared with the in silico hybrid, the range of parental homoeologous expression divergence was substantially reduced in both of the reciprocal hybrids (N9 and 9N) (Kolmogorov–Smirnov test *P* < 2.2e−16), but no significant difference (Kolmogorov-Smirnov test *P* = 0.44) was detected between the reciprocal hybrids (fig. 2*A*). Strikingly, the aggregate range was not only recovered in both reciprocal tetraploids (NN99 and 99NN) but also amplified beyond that shown by the in silico hybrid (Kolmogorov-Smirnov test *P* < 2.2e−16). In this case, a moderate but significant difference (Kolmogorov-Smirnov test *P* < 2.912e−09) was detected between the reciprocal tetraploids (fig. 2*A*). These results indicate that hybridization and WGD have distinctly different impacts on genome-wide expression, due to ploidy level alone. Relative to the in silico hybrid, parental expression divergence is reduced in both reciprocal F1 hybrids, but this suppression was both released and augmented in the reciprocal tetraploids. The lack of difference in gene expression ranges between the two reciprocal hybrids and its existence in the two reciprocal tetraploids suggests a further level of perturbation accompanying polyploidization probably related to direction of parental origin.

Consistent with the suppressed versus expanded ranges for parental expression in the hybrids and tetraploids, respectively, relative to that of the in silico hybrid (fig. 2*A*), the numbers of genes that showed ratio changes of parental expression (compared with the in silico hybrid) differed substantially between the hybrids and tetraploids, with >2,000 more genes showing ratio alteration in the tetraploids (fig. 2*B*). In principle, this indirect comparison for differences between the hybrids and tetraploids mediated by the in silico hybrid would underestimate the effects of WGD. We therefore conducted a direct comparison of homoeologous expression between hybrids and their corresponding tetraploids. We found that in both cross directions approximately 38% of the homoeologs were affected by WGD alone (superimposed on hybridization), and the numbers of affected genes were similar between the two cross directions (4,411and 4,427genes in NN99 vs. N9 and 99NN vs. 9N, respectively) (supplementary fig. S2, Supplementary Material online).

Although in the foregoing analysis we did not find a significant difference in the range of parental expression or the number of affected genes between the reciprocal hybrids and only very small (though statistically significant) difference between the reciprocal tetraploids (fig. 2*A* and *B*), the question remained as to whether or not the same or similar sets of genes were affected in the two pairs of reciprocals. We found that >98% genes were common between the two reciprocal hybrids (only 258 of the 11,608 genes were different) (fig. 2*B*), concordant with the lack of difference between the reciprocal hybrids (fig. 2*A*). In contrast, less than half of the genes were common between the two reciprocal tetraploids (5,882 of the 11,608 genes were different) (fig. 2*B*), far exceeding the magnitude of difference between the two hybrids in levels of expression perturbation. In parallel, in the direct comparison between hybrids and tetraploids, although the numbers of affected genes by WGD were similar between the two cross directions, only 33% (2,202 out of 6,636) of the genes were common (supplementary fig. S2, Supplementary Material online). These results demonstrate that while the suppression of homoeologous expression divergence by hybridization has reproducibly targeted the same set of genes between the two independent crossings with opposite maternal versus paternal origins, subsequent WGD has a far more dramatic and less predictable effect on global gene expression patterns, due either to stochastic (difference due to two independent WGD events irrespective of parent-of-origin) or parent-of-origin effects. Regardless, this finding further points to the fundamentally different impacts of hybridization and WGD on genome-wide homoeologous expression patterns.

To interrogate reliability of our RNA-seq reads calling and analysis, we conducted locus-specific pyrosequencing for a set of 20 homeologous gene pairs (supplementary table S2, Supplementary Material online) that were selected to represent the whole range of expression levels based on the RNA-seq data (see Materials and Methods). We found that the pyrosequencing data were highly correlated with those based on the RNA-seq data (*R*^2 ^= 0.87; supplementary fig. S3, Supplementary Material online), thus validated reliability of the RNA-seq data and the computational methods we used.

### Evolution of *cis*- and *trans*-Regulatory Variation between the Two Rice Subspecies

**Fig. 3.**
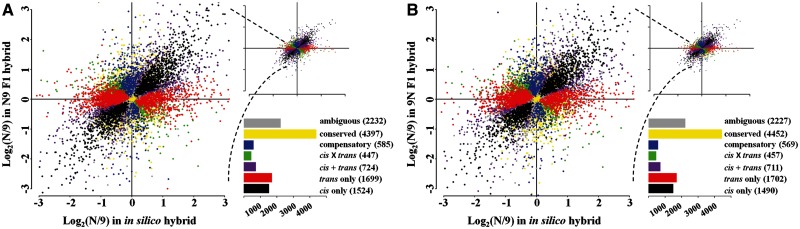
Plot summarizes the relative, homoeolog-specific expression levels in parental (in silico hybrid) and both F1 hybrids. Each point represents a single gene and is color-coded according to the mechanism of regulatory evolution inferred from statistical tests. The bar graph depicts the number of genes in each category.

Expression variation between closely related species can arise from changes in either *cis*- or *trans*-regulation, or both. *cis*-Regulatory sequences have homoeolog-specific effects on gene expression, whereas *trans*-regulatory factors may impact expression of both homoeologs in a hybrid cell. By comparing homoeolog-specific expression within and between the in silico hybrid and the reciprocal hybrids, it is possible to partition expression variation into *cis*- and *trans*-origins, using well-defined criteria (Wittkopp et al. 2004; Springer and Stupar 2007; McManus et al. 2010). Based on these criteria, we classified all 11,608 genes into seven regulatory divergence types according to their expression patterns in the reciprocal hybrids (see Materials and Methods). We found similar results among all regulatory divergence types between the reciprocal hybrids (fig. 3). The *trans-*regulatory divergence type was more abundant than the *cis*-regulatory type in both hybrids, 14.64% (1,699 genes) versus 13.13% (1,524 genes) and 14.66% (1,702 genes) versus 12.84% (1,490 genes) in N9 and 9N, respectively. This relative higher proportion of *trans*- than *cis*-regulatory evolution in the two rice subspecies is different from most previous studies, which were based on smaller numbers of genes (Wittkopp et al. 2004; Springer and Stupar 2007), but is in line with a study in *Drosophila* (McManus et al. 2010) which was based on transcriptomic profiling.

### Convergent versus Divergent Regulation of Homoeologous Expression in Hybrids and Tetraploids

To dissect possible causes at the gene regulation level for the changes in homoeologous expression induced by hybridization versus WGD, we modeled genes into three groups based on their expression patterns in hybrids and/or tetraploids relative to their expression in the in silico hybrid (supplementary fig. S4, Supplementary Material online). Group I is termed “genes under convergent-regulation,” referring to genes for which the relative expression of parental homoeologs in hybrids and/or tetraploids were significantly reduced, relative to the in silico hybrid, changing toward more equal parental expression and approximating a ratio of 1:1. Group II is termed “genes under divergent-regulation” referring to those genes for which the expression divergence of parental homoeologs in hybrids and/or tetraploids were significantly enhanced, relative to a parental expression ratio of 1:1. Group III is termed “genes under conserved-regulation” for which the relative expression of parental homoeologs in hybrids and/or tetraploids were statistically the same as in the in silico hybrid (supplementary fig. S4, Supplementary Material online).

**Fig. 4.**
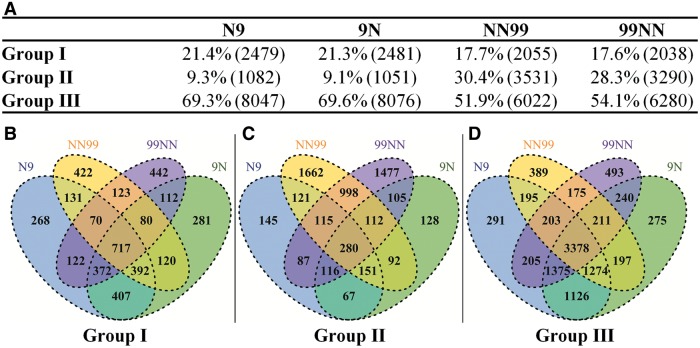
Summary of the numbers of genes belonging to each of the regulatory groups and their relative proportions (*A*), as well as overlaps of the numbers of genes of each of the three regulatory groups between or among all possible comparisons of the four plants, reciprocal hybrids, and reciprocal tetraploids, presented by Venn diagrams (*B–D*).

Results showed that parental expression levels for approximately 70% of the 11,608 analyzed genes showed no difference between the bona fide hybrids and the in silico hybrid (fig. 4*A*); these are Group III genes. The remaining approximately 30% of the genes were significantly different, with respect to homoeolog ratios, from the in silico hybrid, of which about two-thirds belong to Group I (21.4% and 21.3% in N9 and 9N, respectively) and one-thirds to Group II genes (9.3% and 9.1% in N9 and 9N, respectively) (fig. 4*A*). In contrast to the F1 hybrids, in the tetraploids the proportions of conserved genes (Group III) were significantly reduced (to < 55%; χ^2^ test *P* < 2.2e−16), while the proportions of Groups I and II genes together were significantly increased (to >45%; χ^2^ test *P* < 2.2e−16) (fig. 4*A*). Most strikingly, the relative proportions of the Group I versus Group II genes were significantly altered (χ^2^ *P* < 2.2e−16) in tetraploids relative to hybrids; that is, compared with hybrids more genes were under divergent-regulation (Group II) and fewer genes were under convergent-regulation in the tetraploids (fig. 4*A*).

We further dissected the proportions of common and different genes across the two hybrids and two tetraploids in each of the three regulatory gene groups, as shown (fig. 4*B*–*D*). Because the tetraploids were derived from the same hybrid plants, comparisons between hybrids and S1 tetraploids in the same cross direction would reveal the impact of WGD on homoeologous expression without invoking possible parent-of-origin effect. We found that the overlapping proportions between the hybrids and their corresponding tetraploids in either cross direction (Nipponbare × 93-11 or 93-11 × Nipponbare) accounted for >50% of the genes that showed different homoeolog expression in hybrids; we refer to these as “inherited proportions.” The inherited proportions in hybrids of both cross directions of group I are the lowest of the three regulation groups, 52.8% (131 + 70 + 717 + 392; fig. 4*B*) in the N-9 cross direction and 51.6% (112 + 80 + 717 + 372; fig. 4*B*) in the 9-N direction. The Group III genes accounted for the highest proportions of the inherited genes in hybrids of both cross directions, 62.8% (195 + 203 + 3,378 + 1,274; fig. 4*D*) in the N-9 cross direction and 64.4% (240 + 211 + 3,378 + 1,375; fig. 4*D*) in the 9-N direction. The inherited proportions in hybrids in Group II are in the middle but close to Group III, 61.6% (121 + 115 + 280 + 151, fig. 4*C*) in the N-9 cross direction and 58.3% (105 + 112 + 280 + 116, fig. 4*C*) in the 9-N direction. However, the novel proportions in tetraploids in both cross directions are more variable among the three regulation groups. Most Group II genes in the tetraploids are new in both cross directions, which accounted for 81.1% (2,864) in the N-9 cross direction and 81.4% (2,677) in the 9-N direction. Group III contains the fewest number of novel genes in both cross directions of the tetraploids, 16.1% (972) in the N-9 cross direction and 17.1% (1,076) in the 9-N direction. Group I occupies moderate proportions in both cross directions of tetraploids, 36.3% (745) in the N-9 cross direction and 37.1% (757) in the 9-N direction. Collectively, these results show that the tetraploids inherited only small proportion of the genes that already showed altered homoeologous expression in the hybrids but instead were characterized by large numbers of genes that showed novel homoeologous expression.

We performed Gene Ontology (GO) enrichment analysis for all genes of each of the three regulation groups in the hybrids and tetraploids (supplementary table S3, Supplementary Material online). We found several significantly enriched GO categories in both hybrids and tetraploids for Groups I and II genes but none and only one category for Group III genes in hybrids and tetraploids, respectively. In Group I, all enriched categories in tetraploids were also found in hybrids. However, in Group II, some new marginally (*q* values<0.05) enriched categories emerged in tetraploids, including genes involved in epigenetic regulation, cytosol, reproduction, embryo development, and other categories (supplementary table S3, Supplementary Material online).

### Parental Expression Bias in the In Silico Hybrid, Reciprocal F1 Hybrids, and Tetraploids

To quantify the parental expression differences in each sample, we tested the relative expression of each of the 11,608 homoeolog pairs (using the binomial test) against the null hypothesis that homoeologs were equally expressed. To ensure accuracy and reliability of the analysis, we used a FDR method to adjust raw *P* values. Homoeologous pairs with a *q* value <0.05 were regarded as exhibiting biased expression and were further classified into Nipponbare- or 93-11-biased expression.

Among the 11,608 genes harboring diagnostic SNPs between homoeologs, about half (5,823 or 50.2%) showed significantly biased expression by one parental allele in the in silico hybrid. This we regard as reflecting divergent evolution of gene expression that has accumulated in the diploid parental genotypes (Nipponbare and 93-11) representing the two subspecies, *japonica* and *indica*. Consistent with the damping and amplification, respectively, of expression ranges in the hybrids and tetraploids (fig. 2*A*), the number of genes showing biased homoeolog expression was significantly reduced in hybrids (3,977 and 3,898 in N9 and 9N, respectively) but was increased in the tetraploids (6,716 and 6,660 in NN99 and 99NN, respectively), compared with the in silico hybrid (5,823) (supplementary table S4, Supplementary Material online).

To further explore links of biased parental expression in the in silico hybrid with those in the hybrids and tetraploids, we compared all possible categories of gene expression patterns. As illustrated in table 1, the first three categories represent vertical inheritance of parental expression patterns (including both equal and biased expression) by the hybrids and/or tetraploids, which accounted for approximately 50% of all analyzed genes. Notably, tetraploids displayed a drop of about a third (from ∼39% to ∼26%) in the number of genes that retained equal parental expression, relative to the hybrids, whereas for the Nipponbare-biased and 93-11-biased gene categories, frequencies remained similar in the tetraploids and hybrids, though for the latter (93-11 biased), there was a small increase in the tetraploids (table 1). The fourth and fifth categories are genes that showed biased homoeolog expression in the in silico hybrid but were changed to equal expression in hybrids and/or tetraploids. For both of these categories, the proportions in the tetraploids (ranging from 7.68% to 8.43%) were significantly lower (χ^2^ test *P* < 2.2e−16) than those in the hybrids (ranging from 11.38% to 15.57%) (table 1). The sixth and seventh categories represent genes for which homoeologs were equally expressed in the in silico hybrid but showed novel, biased expression in the hybrids and/or tetraploids. For both of these categories, the proportions in the tetraploids (ranging from 10.20% to 13.25%) were significantly higher (χ^2^ test *P* < 2.2e−16) than those in the hybrids (ranging from 4.0% to 6.9%) (table 1). The last two categories represent genes in which the homoeologs showed biased expression in the in silico hybrid but which displayed the opposite directional bias in the hybrids and tetraploids. For both these categories, the proportions in the tetraploids (ranging from 4.67% to 6.23%) were again significantly higher (χ^2^ test *P* < 2.2e−16) than those in the hybrids (ranging from 1.27% to 2.66%) (table 1).

**Table 1.** Biased Homoeologs in the In Silico Hybrid, Reciprocal F1 Hybrids, and Tetraploids.

Expression in Parents^a^	Expression in Hybrids/Tetraploids		N9	9N	NN99	99NN
*N* = 9	*N* = 9	Parental condition	4503 (38.79%)	4545 (39.15%)	3023 (26.04%)	3063 (26.39%)
*N* > 9	*N* > 9	Parental condition	1297 (11.17%)	1249 (10.76%)	1322 (11.39%)	1253 (10.79%)
*N* < 9	*N* < 9	Parental condition	951 (8.19%)	952 (8.20%)	1357 (11.69%)	1497 (12.90%)
*N* > 9	*N* = 9	No bias in hybrids/tetraploids	1321 (11.38%)	1368 (11.78%)	891 (7.68%)	933 (8.04%)
*N* < 9	*N* = 9	No bias in hybrids/tetraploids	1807 (15.57%)	1797 (15.48%)	978 (8.43%)	952 (8.20%)
*N* = 9	*N* > 9	Novel bias in hybrids/tetraploids	801 (6.90%)	776 (6.69%)	1403 (12.09%)	1184 (10.20%)
*N* = 9	*N* < 9	Novel bias in hybrids/tetraploids	481 (4.14%)	464 (4.00%)	1359 (11.71%)	1538 (13.25%)
*N* > 9	*N* < 9	Opposite bias in hybrids/tetraploids	147 (1.27%)	148 (1.27%)	552 (4.76%)	579 (4.99%)
*N* < 9	*N* > 9	Opposite bias in hybrids/tetraploids	300 (2.58%)	309 (2.66%)	723 (6.23%)	609 (5.25%)

Note.—*N* = 9 denotes equal expression; *N* > 9 and *N* < 9 denote Nipponbare-biased and 93-11-biased expression, respectively.

^a^Based on the homoeolog expression bias test in the in silico hybrid.

Taken together, it is clear that both hybrids and tetraploids of rice subspecies manifested dramatically altered parental expression biases relative to the in silico hybrid. However, fundamental differences existed between the two plant groups, hybrids and tetraploids. While more genes are under convergent regulation in hybrids relative to the in silico hybrid, the opposite is true in tetraploids, in which the expression of more genes are subjected to divergent regulation.

## Discussion

It has been suggested that the differential impacts of hybridization and WGD on genome-wide gene expression and their attendant biological consequences may play an important role in the evolutionary trajectories of nascent allopolyploids toward either stabilization and speciation or extinction (Mayrose et al. 2011). Consistent with this possibility, the impacts of hybridization versus WGD on gene expression changes, both with regard to their relative importance and characteristics, appear to be highly idiosyncratic among plant taxa (Hegarty 2011).

Gene expression regulation entails *cis*- and *trans*-acting components and their myriad synergistic and antagonistic interactions (Wittkopp et al. 2004; McManus et al. 2010). In hybrids or allopolyploids, diverged parental regulatory components are introduced into a common nucleus and cytoplasm, whereby the *cis*- and *trans*-regulatory factors from different parental species may interact to cause up- or downregulation of one or both parental homoeologs for a given gene (Yoo et al. 2013), which observationally is revealed as transcriptomic shock (Hegarty et al. 2006; Buggs et al. 2011). Experimentally, plant systems encompassing reciprocally generated hybrids and polyploids from the same pure-line parental lines are ideal for addressing the differential impacts of hybridization and WGD and for teasing apart *cis*- and *trans*-effects. Here, we report construction of such a model system using two sequenced genotypes of Asian rice (*Oryza sativa* L.) representing its two subspecies, *japonica* and *indica*. Although sharing a common wild progenitor (*O. rufipogon*) and possibly experiencing reciprocal introgressions, the two subspecies harbor extensive genic, genomic, and functional divergence due to strong human-mediated selection for agronomic performance under distinct ecological conditions (Zhang et al. 2009). Thus, hybrids and polyploids generated from the two subspecies are suitable for exploring the distinct impacts of hybridization and WGD on gene expression and their biological consequences. Indeed, reciprocal hybrids of the two rice subspecies have been used extensively as a model to investigate the molecular basis of heterosis. These studies show that genome-wide alterations of gene expression, including biased parental expression, occurred in the hybrids, resulting from both inherited genetic and epigenetic divergence primarily at gene promoter regions and de novo epigenetic remodeling in the hybrids (He et al. 2010; Ouyang et al. 2010; Chodavarapu et al. 2012). Hybrid plants are sterile, but we found that high fertility was restored in the tetraploids, which, together with their largely stable euploid nature, renders the synthetic plants suitable for evolutionary studies.

The significantly altered phenotypes in the tetraploids relative to the hybrids sharing the same parentage constitute de facto phenotypic evidence of the differential impacts of hybridization and WGD on gene expression. With respect to hybridization, the most conspicuous transcriptomic effect, shown here, is to reduce divergent parental expression differences that accumulated during divergence of the two diploid parents from their common wild ancestor, *O. rufipogon* (Kovach et al. 2007), and/or inherited standing genetic polymorphisms of different accessions of the wild progenitor involved (Huang et al. 2012).

A useful framework for interpreting these results is provided by the idea that divergent *cis*- and *trans*-factors inherited from the parental lines in the common hybrid cellular context would interact (Wittkopp et al. 2004; Chaudhary et al. 2009; Tirosh et al. 2009; McManus et al. 2010; Shi et al. 2012). Presumably, divergent evolution at the diploid level between the parental lines generated diverged *cis*- and *trans*-regulatory factors throughout the genome. When these diverged factors are combined into a single nucleus, transcription of two, now-divergent alleles will be controlled not only by native *cis*-regulatory enhancers and promoters but by novel *trans*-acting factors (e.g., transcription factors or chromatin modifiers). As a result, parental expression differences may be reduced or eliminated in the hybrids for genes subjected to strong *trans*-regulation; this is detected globally as a reduced range of parental expression difference in the hybrids relative to that of the in silico hybrid. This scenario is compatible with the observation of the higher proportion of genes under convergent-regulation (Group I genes) and lower proportions of genes under divergent-regulation (Group II genes) in the hybrids compared with the in silico hybrid (fig. 2*A*).

Although the *cis*/*trans* framework provides a satisfying explanation for globally observed reduction in allelic expression differences in the F1 hybrids, it is less obviously applicable to our observations for the two reciprocal allotetraploids (fig. 2*A*). In this case, expression of the two divergent homoeologous is dramatically augmented as a consequence of WGD, in many cases at levels beyond the ranges of the parents and the in silico hybrid. Consistent with this, the proportions of genes under divergent-regulation (Group II genes) are increased to a much greater extent than is the decrease in genes under convergent-regulation (Group I genes) in the tetraploids relative to the hybrids. We note that this conspicuous increase primarily is due to two categories of genes, for which homoeologous expression is transgressive in the tetraploids compared with the in silico hybrid and hybrids. These genes are those that showed biased expression between the homoeologs in tetraploids but which are expressed equally in the in silico hybrid and actual hybrids, and those that showed biased expression in tetraploids but with opposite directions from their biased expression in the in silico hybrid and hybrids.

How might we account for this dramatic difference in allelic (at the diploid level) and homoeologous (at the allotetraploid level) gene expression for two entities that share identical suites of *cis*- and *trans*-regulatory factors? Clearly the answer has ploidy level itself as its root cause, as this is the only known difference among the plants studied. One possibility, however, is the operation of one or more of the myriad genetic and epigenetic alterations that previously have been shown to characterize rapid de novo evolution in allopolyploids. This suite of phenomena includes genetic changes such as gene loss, rearrangement, recombination, and gene conversion (Ozkan et al. 2001; Salmon et al. 2010; Szadkowski et al. 2011), and/or epigenetic remodeling (Madlung et al. 2002; Lukens et al. 2006; Parisod et al. 2009; Zhao et al. 2011) and/or interaction and/or differential titration of parental small RNAs (Ha et al. 2009; Kenan-Eichler et al. 2011). While these various mechanisms may be involved, an alternative hypothesis seems more likely, namely, that genes that differ in *cis*-acting genetic factors and epigenetic states (He et al. 2010; Ouyang et al. 2010; Chodavarapu et al. 2012) may differ in their regulatory sensitivity to WGD due to perturbations of gene balance (Stupar et al. 2007; Riddle et al. 2010; Yu et al. 2010; Birchler and Veitia 2012; Sun et al. 2013; Yao et al. 2013). We speculate that these disruptions in gene balance are mechanistic consequences of quantitative or stoichiometric effects that propagate through the transcriptomic network due to alterations in chromatin packaging, nuclear and cellular volumes, and the many other differences that arise when a cell saltationally acquires a doubling in its chromosome number. It should be noted that given the relative closer genetic relationship between the two parental subspecies in our case compared with interspecific combinations for most other allopolyploid plants studied (e.g., wheat and cotton), recombinational loss and homoeologs may occur continuously in the rice tetraploids in successive generations, as in *Tragopogon* (Buggs et al. 2012), which may cause additional homeologous expression divergence. However, even without any changes to genic content or *cis*- or *trans*-regulatory controls, the emergent property of “whole genome doubling” has repercussions that reverberate throughout the transcriptome, and we propose, throughout the proteome and higher levels of cellular organization, to ultimately yield dramatically altered phenotypes. Further insight into this idea will derive from future studies, particularly those that are imbued with a network perspective and which incorporate improved methods for characterizing higher levels of “omics” that lead to phenotype. Ultimately, this perspective may yield insight into the nature of adaptation and the origin of evolutionary novelty accompanying polyploidy.

## Materials and Methods

### Plant Materials

Reciprocal F1 hybrids N9 and 9N were produced by crossing the two fully sequenced cultivars (Nipponbare and 93-11) representing the two subspecies, *japonica* and *indica*, of Asian rice (*Oryza sativa* L.). Half of the tillers of the F1 hybrid plants were separated, planted into independent pots, and treated with colchicine to induce whole genome doubling (WGD), while the other half (hybrids) were used for tissue collection. In this manner, the tetraploids and hybrids were generated from exactly the same plants. Spikes showing dramatically improved seed setting were harvested. The seeds were germinated and root tips were taken for cytological observations by conventional methods. All doubled plants (designated as S1) with a confirmed euploid chromosome number (2*n* = 48) were used for tissue collection. All plants, including Nipponbare, 93-11, N9, 9N, NN99, and 99NN, were grown under the same controlled growing condition optimized for rice in greenhouse. Young panicles (∼0.5 cm in length), harvested from three individuals of each genotype as pools, were immediately frozen in liquid nitrogen before being stored at −80 °C until use.

### RNA Extraction and Hiseq2000 Sequencing

Total RNAs were isolated from the frozen tissues using Trizol (Invitrogen). Integrity of RNA samples was determined using an Agilent 2100 Bioanalyzer (Agilent Technologies, Waldbronn, Germany) according to the supply's specifications. Library construction, cluster generation, and Hiseq2000 sequencing were carried out with standard protocols. All samples were treated and sequenced as parallel experiments and for each genotype one library with barcode was sequenced in the same Hiseq2000 run. Raw data were cleaned by removing adaptor contamination and low-quality reads. In total, we obtained more than 31 billion high-quality nucleotides (∼170 million pairs of 90 nt reads). For each reciprocal hybrids and tetraploids, approximately 6 Gb sequences were obtained. Approximately 3.5 Gb sequences were acquired for each parental samples 93-11 and Nipponbare (supplementary table S5, Supplementary Material online). Clean data have been deposited at the SRA database http://www.ncbi.nlm.nih.gov/sra/ (last accessed March 11, 2014) with accession number SRP035332.

### Identification of SNPs between Parental Homoeologs

The 93-11 genome sequence (Yu et al. 2002) was obtained from RISe (Rice Information System, http://rice.genomics.org.cn, last accessed March 11, 2014; latest version) and was usedto simulate 50 million 100 bp paired end genomic reads by the “dwigsim” program (http://sourceforge.net/apps/mediawiki/dnaa/index.php?title = Whole_Genome_Simulation, last accessed March 11, 2014). All simulated 93-11 genomic DNA reads were mapped to the Nipponbare reference genome sequence (Goff et al. 2002; International Rice Genome Sequencing Project 2005) at MSU7.0 (http://rice.plantbiology.msu.edu/, last accessed March 11, 2014) by the BWA program (Li and Durbin 2009). A SNPs list between 93-11 and Nipponbare genome DNA sequences were generated by the Genome Analysis Toolkit (GATK) (McKenna et al. 2010; DePristo et al. 2011). The RNA-seq data of Nipponbare and 93-11 were then mapped to the Nipponbare reference genome using the TopHat software (-N 4, –read-edit-dist 4, –no-novel-juncs). First, alignments from Nipponbare (N) and 93-11 (9) were used to detect SNPs in transcript regions between the two homoeologs of each gene using samtools (Li et al. 2009) and custom perl programs. To insure reliability of homoeologous transcript SNPs, for 93-11 RNA-seq data, we only selected those sites falling into a consensus exon with a depth >10 contingent with the alternative bases (SNP bases) accounting for >95% of the reads mapped to the site. For Nipponbare RNA-seq data, we also acquired a SNPs list with similar criteria but the alternative bases (SNP bases) were accounting for >5% of the reads mapped to a given site, which accounted for the variations between our plants and the reference genome as well as mapping as sequencing errors. Finally, only the SNPs found in both 93-11 RNA-seq data and previous genomic DNA data, but not included in Nipponbare RNA-seq data, were used in further homoeologous expression analysis.

### Estimation of Relative Homoeologous Expression

Cleaned data of all samples were mapped by TopHat (version 2.0.0) (Kim et al. 2013) against the updated Nipponbare genome (MSU7.0 http://rice.plantbiology.msu.edu/, last accessed March 11, 2014) in which all known genomic SNPs produced by previous procedure were replaced by IUPAC bases. The .bam files generated by Tophat were transferred to pileup format using samtools (version 0.1.17) (Li et al. 2009). A series of custom Perl programs were used to tabulate read counts containing either of the parental SNPs across the whole genome. Relative homoeologous expression for MSU7.0 gene models was calculated by comparing parental read numbers containing corresponding SNPs.

### Locus-Specific Pyrosequencing

To evaluate reliability of the computational methods used, we tested the relative homoeologous expression of a subset of 20 representative homoeologous gene pairs (supplementary table S2, Supplementary Material online) by locus-specific cDNA pyrosequencing (Zhang et al. 2013) on all plant samples using the same RNAs as for RNA-seq by PyroMark (Biotage AB, Sweden). Briefly, cDNA prepared from each sample was used for gene-specific PCR amplifications with primers designed at fully conserved regions between the parental homoeologs, followed by pyrosequencing reactions to determine relative expression ratios of parental homoeologs. Twenty representative genes with various levels of homoeologous expression according to the RNA-seq data (supplementary table S2 and fig. S3, Supplementary Material online) were selected for pyrosequencing analysis. Correlation between the two sets of ratios of homoeologous expression based on the RNA-seq data and pyrosequencing was calculated by linear regression analysis.

### Assignment of *cis*- and *trans*-Regulatory Divergence

The *cis*- and *trans*-regulatory divergence assignments were carried out by using the procedures described previously (McManus et al. 2010). Parental homoeolog (or allele) representations in the in silico hybrid (P) and reciprocal F1 hybrids N9 and 9N (H) were analyzed for evidence of differential homoeologous expression using the binomial exact test (homoeolog expression 1:1 test), with raw *P* values corrected by FDR method. Any significant difference in the abundance of Nipponbare and 93-11 homoeologs in the P data set was considered as evidence of parental expression divergence, and any significant difference in the abundance of Nipponbare and 93-11 homoeologs in the H data set was considered evidence of *cis*-regulatory divergence. Genes found to be differentially expressed in either the P or H data sets were further analyzed for *trans* effects (T) by comparing parental-specific transcript abundance ratios between the P and H samples using χ^2^ exact tests with a Yates correction, raw *P* values were corrected by FDR method. Based on these criteria (McManus et al. 2010), all 11,608 genes were categorized into the following seven types:1) *cis* only: significant differential expression in P and H, but not significant in T. 2) *trans* only: significant differential expression in P and T, but not in H. 3) *cis* + *trans*: significant differential expression in P, H, and T. Moreover, the relative expression between the parental homoeologs (*N* > 9 or *N* < 9) of these genes in the in silico hybrid and the true F1 hybrids is the same; that is, the parental homoeologs with higher expression in the in silico hybrid also showed higher expression than the other homoeologs in F1 hybrids and vice versa. Apparently, the regulation of these genes has diverged in such a way that *cis*- and *trans*-regulatory differences favor expression of the same parental homologs. 4) *cis* × *trans*: significant differential expression in P, H, and T. Nonetheless, the relative expression between the parental homoeologs (*N* > 9 or *N* < 9) of these genes in the in silico hybrid and the true F1 hybrids is opposite; that is, the parental homoeologs with higher expression in the in silico hybrid showed lower expression than the other homoeologs in F1 hybrids and vice versa. The regulation of these genes has diverged in such a way that *cis*- and *trans*-regulatory differences favor expression of opposite homoeologs. 5) Compensatory: significant differential expression in H, but not in P. Significant T. Regulation of these genes has diverged in such a way that *cis*- and *trans*-regulatory differences perfectly compensate each other, resulting in no expression difference between the parental homoeologs. 6) Conserved: no significant differential expression in H or P. No significant T. These genes are expressed at a similar level between the parental homoeologs, as well as in the true hybrid, indicating conserved regulation. 7) Ambiguous: all other patterns of significance tests, which have no clear biological interpretation.

### GO Enrichment Analysis

To assay for possible differential functional relevance of the genes categorized into each of the three regulation groups, we performed GO enrichment analysis with GO Slim assignments from MSU7.0 (http://rice.plantbiology.msu.edu/, last accessed March 11, 2014). All GO terms containing genes from any regulatory groups were calculated by hypergeometric test, and raw *P* values were adjusted by FDR method to calculate *q* values. Only GO terms with *q* value <0.05 were regarded as significantly enriched.

### Statistics

All statistical and graphical analyses were carried out in the *R* computing environment (version 2.11.1). For the identification of three regulation groups in hybrids and polyploids, first reciprocal hybrids and tetraploids were compared with the in silico hybrid by χ^2^ test (prop.test in *R*) followed by FDR corrections, respectively. Significantly different genes (*q* value < 0.05) in every hybrid and tetraploid sample were subjected to further comparative analysis of divergence (absolute distance to equal expression) to the in silico hybrid for different regulation patterns. To assay homoeologous expression bias, parental expression ratios for all genes were tested using the binominal test with the null hypotheses that parental homoeologs were equally expressed; raw *P* values were adjusted by FDR method. Any gene with a *q* value <0.05 was considered as significantly different from equal expression of Nipponbare and 93-11 homoeologs and classified as a gene with biased expression.

## Supplementary Material

Supplementary materials, tables S1–S5, and figures S1–S4 are available at *Molecular Biology and Evolution* online (http://www.mbe.oxfordjournals.org/).

Supplementary Data
