# Supplementary material for: Genome-Wide Disruption of Gene Expression in Allopolyploids but Not Hybrids of Rice Subspecies
Source: Mol Biol Evol. 2014 Feb 27;31(5):1066–76. doi: 10.1093/molbev/msu085 (PMC3995341; doi:10.1093/molbev/msu085)
Supplement: Supplementary Data [file supp_31_5_1066__index.html]

Genome-Wide Disruption of Gene Expression in Allopolyploids but Not Hybrids of Rice Subspecies — Genome-Wide Disruption of Gene Expression in Allopolyploids but Not Hybrids of Rice Subspecies — Supplementary Data 

# Genome-Wide Disruption of Gene Expression in Allopolyploids but Not Hybrids of Rice Subspecies

## Supplementary Data

files

**Files in this Data Supplement:**

- Supplementary Data - pdf file
- Supplementary Data - pdf file
- Supplementary Data - xlsx file
- Supplementary Data - xlsx file
