# Supplementary material for: Genome-Wide Disruption of Gene Expression in Allopolyploids but Not Hybrids of Rice Subspecies
Source: Mol Biol Evol. 2014 Feb 27;31(5):1066–76. doi: 10.1093/molbev/msu085 (PMC3995341; doi:10.1093/molbev/msu085)
Supplement: Supplementary Data [file supp_msu085_FIG_S1-S4.pdf]

Supplementary Material

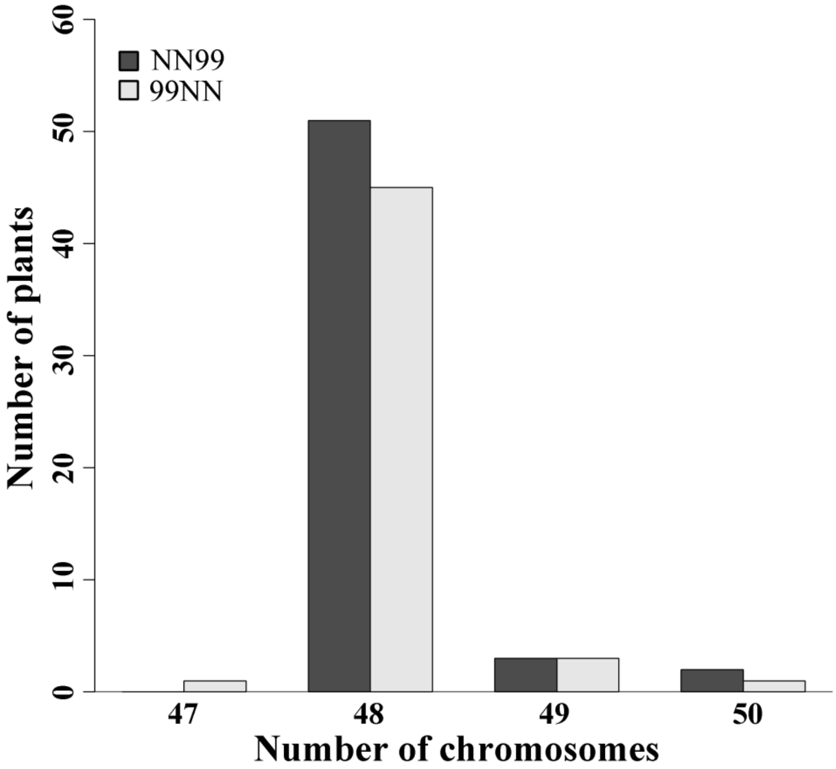

FIG. S1. Summary of chromosome number distribution in the reciprocal tetraploids derived from the corresponding reciprocal F1 hybrids of the two subspecies of rice, *japonica* (cv. Nipponbare) and *indica* (cv. 93-11)

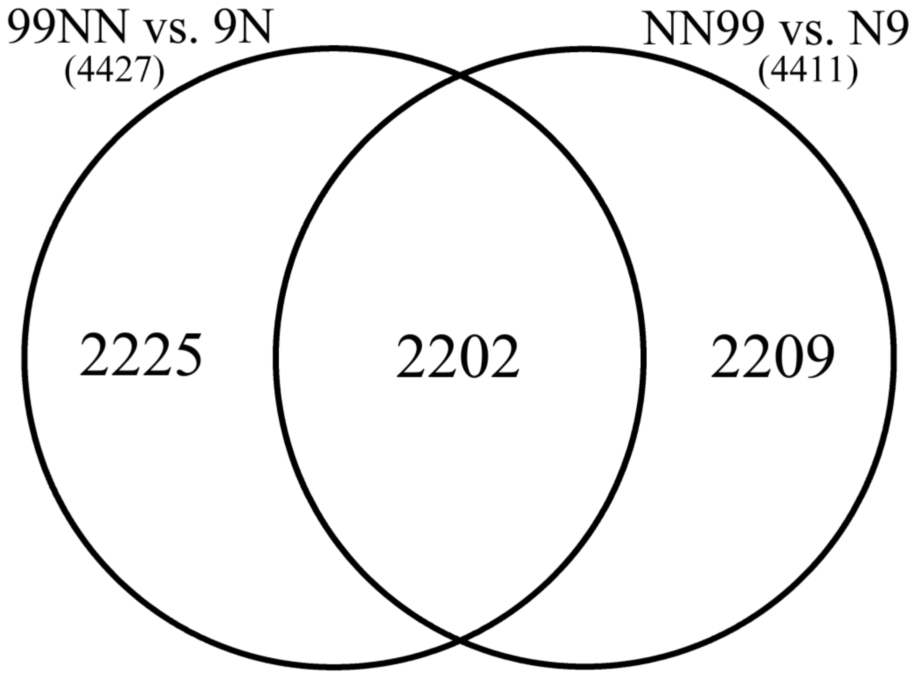

FIG. S2. Venn-diagram of differentially regulated genes through a direct comparison of homoeologous expression between hybrids and their corresponding tetraploids of both cross directions.

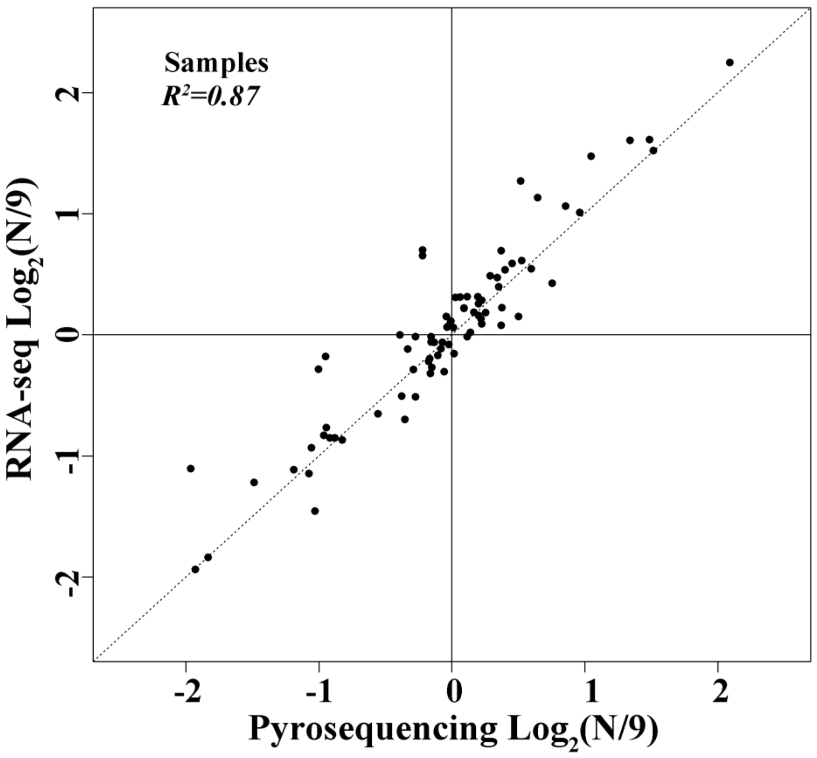

FIG. S3. Correlation between data of pyrosequencing and RNA-seq on parental homoeolog expression ratios for 20 representative genes

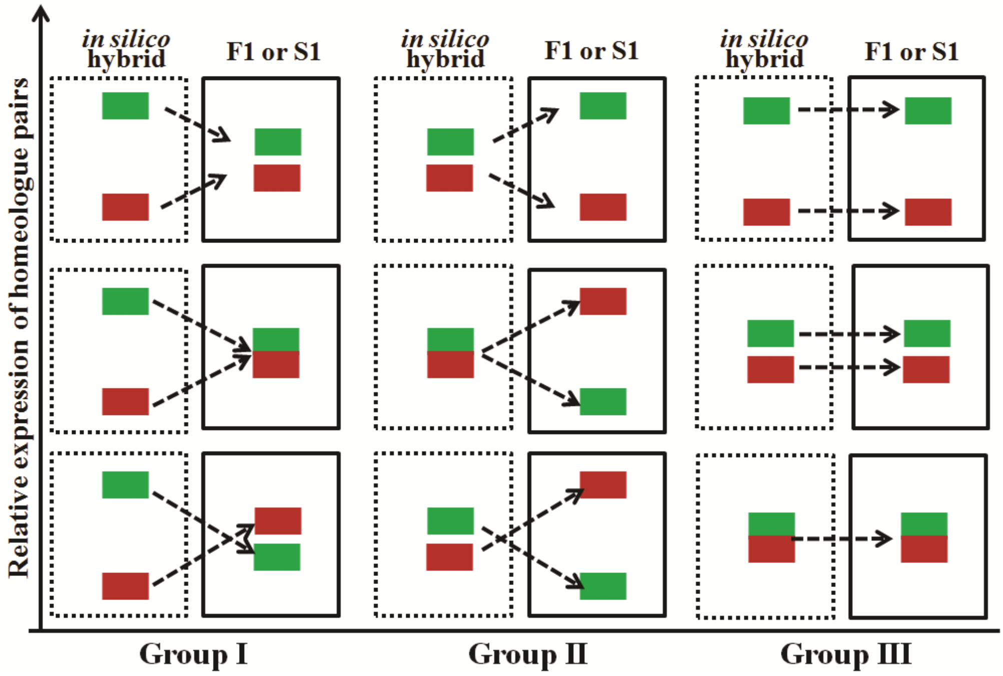

FIG. S4. Diagrammatic illustration of the three regulatory groups of gene expression, convergent-regulation (Group I), divergent regulation (Group II) and conserved-regulation (Group III) that may occur in the F1 hybrids and tetraploids compared with the *in silico* hybrid.
