# Supplementary material for: Genome-Wide Disruption of Gene Expression in Allopolyploids but Not Hybrids of Rice Subspecies
Source: Mol Biol Evol. 2014 Feb 27;31(5):1066–76. doi: 10.1093/molbev/msu085 (PMC3995341; doi:10.1093/molbev/msu085)
Supplement: Supplementary Data [file supp_msu085_TABLE_S2_S4_and_S5.pdf]

Table S2. Pyrosequencing validation of RNA-seq results of parental homoeolog expression ratios (Nipponbare/9311) in the reciprocal hybrids (N9 and 9N) and tetraploids (NN99 and 99NN) for 20 genes representative of the range of gene expression levels in rice

	N9		9N		NN99		99NN	
Gene	Pyro-seq	RNA - seq	Pyro-seq	RNA - seq	Pyro-seq	RNA - seq	Pyro-seq	RNA - seq
LOC_Os03g10800	0.50	0.82	0.52	0.88	0.93	0.89	0.26	0.46
LOC_Os02g44870	1.17	1.06	1.16	1.09	0.89	0.86	0.54	0.55
LOC_Os02g45070	0.90	0.83	0.96	0.81	0.77	0.70	0.49	0.36
LOC_Os02g08120	1.15	1.12	1.42	1.11	1.29	1.06	0.78	0.62
LOC_Os01g42260	1.15	1.24	1.17	1.22	2.07	2.78	1.56	2.19
LOC_Os07g38730	1.16	1.21	1.19	1.14	1.51	1.46	2.80	3.06
LOC_Os09g20350	1.07	1.16	1.07	1.16	0.89	0.87	1.69	1.34
LOC_Os01g18670	0.89	0.80	1.01	0.90	4.26	4.75	1.27	1.39
LOC_Os07g43870	0.90	0.99	0.90	0.96	2.53	3.05	1.22	1.40
LOC_Os08g06010	1.08	1.24	1.12	1.14	0.48	0.52	2.86	2.87
LOC_Os01g01700	1.02	1.24	1.04	1.24	1.44	1.53	0.44	0.46
LOC_Os09g24440	1.37	1.50	1.29	1.62	0.80	0.92	0.52	0.59
LOC_Os05g23860	1.32	1.45	1.28	1.32	0.47	0.45	1.30	1.17
LOC_Os03g56270	1.01	1.04	0.99	1.06	1.15	1.19	1.95	2.01
LOC_Os08g22354	1.00	1.08	0.95	0.92	0.56	0.55	1.10	1.01
LOC_Os05g45930	0.95	0.96	0.76	1.00	0.28	0.28	1.81	2.09
LOC_Os03g10340	1.08	0.99	0.91	0.96	0.98	0.95	0.53	0.55
LOC_Os10g39760	0.83	0.99	0.82	0.82	0.26	0.26	0.83	0.70
LOC_Os10g30100	0.98	1.04	0.97	1.11	0.51	0.56	0.68	0.64
LOC_Os05g45050	0.86	1.62	0.86	1.57	0.36	0.43	1.43	2.41

Table S4. Summary of biased homoeologs in the *in silico hybrid,* the reciprocal hybrids (N9 and 9N) and tetraploids (NN99 and 99NN)

	in silico hybrid	N9	9N	NN99	99NN
N > 9	2,765	2,398	2,334	3,448	3,046
N < 9	3,058	1,579	1,564	3,268	3,614
Total	5,823	3,977	3,898	6,716	6,660

Table S5. Summary of RNA-seq clean data

Samples	Total Reads	Total Nucleotides	Q20 Percentage
Nipponbare	39,297,824	3,536,804,160	96.93%
9311	38,674,310	3,480,687,900	97.13%
N9	68,450,232	6,160,520,880	96.99%
9N	68,354,908	6,151,941,720	96.88%
NN99	67,917,614	6,112,585,260	96.83%
99NN	64,825,692	5,834,312,280	96.93%
